# Knowledge and practices regarding toxoplasmosis in housewives: A cross sectional study in a northern Mexican city

**DOI:** 10.1371/journal.pone.0222094

**Published:** 2019-09-09

**Authors:** Nadia Velázquez-Hernández, Ana Yuliana Avilés Ávila, Manuel Arturo Rivas-González, Selma Paola Delgado-González, Gustavo Alexis Alvarado-Félix, Ángel Osvaldo Alvarado-Félix, Isabel Beristain-Garcia, Cosme Alvarado-Esquivel

**Affiliations:** 1 Institute for Scientific Research “Dr. Roberto Rivera Damm”, Juárez University of Durango State, Durango, Mexico; 2 Facultad de Enfermería y Obstetricia, Juárez University of Durango State, Durango, Mexico; 3 Unidad de Medicina Familiar No. 49, Instituto Mexicano del Seguro Social, Durango, Mexico; 4 Colegio Anglo-Español Durango, Durango, Mexico; 5 Faculty of Medicine and Nutrition, Juárez University of Durango State, Durango, Mexico; Clemson University, UNITED STATES

## Abstract

We aimed to determine the knowledge and practices regarding toxoplasmosis among housewives in the northern Mexican city of Durango. One hundred eighty-five women (mean age: 41.27 ± 12.40 years old) with an occupation of housewife were studied. A self-administered questionnaire was used. This tool included items about the parasite *Toxoplasma gondii*, its transmission routes, general clinical, diagnostic, and treatment aspects of toxoplasmosis, and practices to avoid infection. A minority (<10%) of women knew about the parasite, the disease, how the transmission occurs, the clinical manifestations, how an infection is diagnosed, the treatment, and how to avoid toxoplasmosis. Some women knew that cats can transmit *T*. *gondii* infection (20%), and that the parasite can be found in cat feces (20.5%). Only 7.6% of women knew that infection with *T*. *gondii* can be transmitted by consumption of contaminated food or water. Only 1.1% of women knew about the prevalence of *T*. *gondii* infection. Some (4.9%) women used to taste raw meat while cooking, and 7.6% used to undercook meat. In addition, 20% of women used to eat raw dried meat, and 13.5% consumed untreated water. Less than 90% of women always washed their hands before cooking, and washed fruits or vegetables. The majority (75.1%) of women never wore gloves when handling raw meat. About one quarter (27.6%) of women always froze meat. And 16.2% of women cleaned cat feces. This is the first study regarding knowledge and practices about toxoplasmosis in housewives. Poor knowledge regarding *T*. *gondii* infection, toxoplasmosis, and practices to avoid infection among the housewives studied was found. High risk practices for infection were identified. Strategies to improve toxoplasmosis-related knowledge and practices to avoid *T*. *gondii* infection and its sequelae in housewives are highly needed.

## Introduction

Infections with the parasite *Toxoplasma gondii* (*T*. *gondii*) are common in humans and animals around the world [1]. Nearly one-third of humanity has been exposed to this parasite [2]. Infection in humans usually occurs by consumption of meat from infected animals or by ingestion of water or food contaminated with feces from infected cats [3, 4]. Soil is a source of *T*. *gondii* infection [5]. Raw or undercooked meat from *T*. *gondii*-infected warm-blooded animals is an important source of infection for humans [6, 7]. Infection with *T*. *gondii* can also occur by blood transfusion [8], or organ transplant [9]. Congenital infection with *T*. *gondii* may occur when a primary infection is present in a pregnant woman leading to miscarriage, fetal death, and neurological, ocular, or other damage in the fetus [10]. Sexual transmission of *T*. *gondii* might occur and this could have an impact on the risk of congenital toxoplasmosis [11]. Infection with *T*. *gondii* is usually asymptomatic but may lead to chorioretinitis and encephalitis mainly in immune deficiency conditions [12]. In addition, infection with *T*. *gondii* may cause lymph node enlargement [13]. Serological diagnosis is used routinely to determine the immune status for infection with *T*. *gondii* [14]. There is treatment for *T*. *gondii* infection, but failure rates remain significant [15].

To the best of our knowledge, this is the first study regarding knowledge and practices about toxoplasmosis in housewives. Toxoplasmosis is a food-borne disease [16], and thus persons who prepare food as housewives may play an important epidemiological role to avoid *T*. *gondii* infection. In addition, they teach sanitary measures to their children. Therefore, in this study we sought to determine the knowledge and practices regarding toxoplasmosis in housewives in the northern Mexican city of Durango. Information obtained in this study may help for an optimal planning of education on prevention against toxoplasmosis and its sequelae in housewives and their families.

## Materials and methods

### Study design and women studied

Through a cross sectional study design, we surveyed women with housewife occupation in Durango City, Mexico. Inclusion criteria for enrollment of women were: 1) women with housewife occupation; 2) aged 18 years and older; 3) who voluntarily accepted to participate in the study. Socioeconomic status was not a restrictive criterium for enrollment. As a strategy to enroll women, they were invited to participate by visiting them at their homes (n = 63), when attended a clinic for family planning (n = 83), and in a school when they picked their children up (n = 39). In total, 185 women were studied form July to December 2018.

### Knowledge and practices about toxoplasmosis

We designed a questionnaire to obtain the sociodemographic characteristics, knowledge and practices about toxoplasmosis in the study population. The questionnaire was self-administered and anonymous. All items about knowledge and practices of toxoplasmosis were multiple choice questions. Questionnaire items were developed considering basic knowledge about the parasite and the disease. We included epidemiological aspects of infection as magnitude of the infection (prevalence), hygiene practices, transmission routes, prevention, diagnosis, and treatment. The questionnaire was written in plain language, considering that the wording, structure, and design were clear for the housewives. We reviewed several times the questionnaire and changed the wording when needed to ensure that the readers could understand all questions. Sociodemographic characteristics included age, birthplace, residence, educational level, and socioeconomic status. Items about knowledge and practices of toxoplasmosis included questions regarding the infectious agent, transmission routes of *T*. *gondii* infection, diagnosis, clinical manifestations, prevalence, prevention, and treatment of toxoplasmosis. Questions about the practices included seniority in the occupation, number of persons eating from the meal she cooks, raw meat tasting, degree of meat cooking, consumption of raw dried meat, untreated water, unwashed fruits or vegetables, washing hands before cooking, wearing gloves when handling raw meat, washing knife after used for raw meat cutting, freezing meat, animal contact, and cleaning cat feces. We summitted the first 50 questionnaires to housewives and observed whether they understood all items. None of the subjects reported a problem in understanding the items of the questionnaire. Thus, we continued submitting the questionnaires, and at the end of the survey, no problem in understanding the questions or answers was reported. The Spanish and English versions of the questionnaire are available (S1 and S2 Files, respectively).

### Statistical analysis

Data was analyzed with the software SPSS version 20 using descriptive statistics. Epi Info version 7 was used to calculate the sample size. For this purpose, we used: a) 11% as the expected frequency for the factor under study [17], b) 100000 as the population size (housewives) from which the sample was selected, c) 5% of confidence limits, d) a design effect of 1.0, e) one cluster, and f) a 95% confidence level. The result of the sample size calculation was 150 subjects.

### Ethics aspects

This project was approved by the Ethical Committee of the Faculty of Medicine and Nutrition of the Juárez University of Durango State. Participation was voluntary. The Ethics Committee indicated no need to obtain a written informed consent in this survey. The questionnaire was anonymous; therefore, any document as a written informed consent that might reveal the identity of the subjects was asked. However, subjects provided a verbal consent to participate in the study, and people accompanying them including relatives, nurses, or teachers witnessed.

## Results

Studied women were 20 to 78 (mean: 41.27 ± 12.40) years old. Most of them were born in Durango State, Mexico (89.2%), resided in urban areas (88.1%), and had a medium socioeconomic status (94.1%). Details of the sociodemographic data of the studied women are shown in Table 1.

**Table 1:** General characteristics of the population studied.

Characteristic	No.	%
Age (years old)		
≤30	41	22.2
31 to 50	102	55.1
> 50	42	22.7
Birthplace		
Durango	165	89.2
Other Mexican State	19	10.3
Other country	1	0.5
Residence place		
Durango	180	97.3
Other Mexican State	3	1.6
Abroad	2	1.1
Residence area		
Urban	163	88.1
Suburban	12	6.5
Rural	10	5.4
Education level		
No education	6	3.2
1 to 6 years	15	8.1
7–12 years	102	55.1
>12 years	62	33.5
Socio-economic level		
Low	10	5.4
Medium	174	94.1
High	1	0.5

Concerning knowledge about toxoplasmosis, a minority (<10%) of studied women knew about the parasite, the disease, how the transmission occurs, the clinical manifestations, treatment, how an infection is diagnosed, and how to avoid toxoplasmosis. Details of the knowledge found in the studied women are shown in Table 2. Some women knew that cats can transmit *T*. *gondii* infection (20%), and that the parasite can be found in cat feces (20.5%). Only 7.6% of women knew that infection with *T*. *gondii* can be transmitted by consumption of contaminated food or water. Only 1.1% of women knew about the prevalence of *T*. *gondii* infection.

**Table 2:** Knowledge about *Toxoplasma gondii* and epidemiological, and clinical aspects of toxoplasmosis.

Questions and answers	No.	%
Do you know what is *Toxoplasma gondii*?		
Yes	18	9.7
No	167	90.3
Do you know what toxoplasmosis is?		
Yes	14	7.6
No	171	92.4
Do you know how *Toxoplasma gondii* is transmitted?		
Yes	18	9.7
No	167	90.3
Do you know the clinical manifestations of toxoplasmosis?		
Yes	11	5.9
No	174	94.1
Do you know how *Toxoplasma* infection is diagnosed?		
Yes	9	4.9
No	176	95.1
Do you know how to avoid toxoplasmosis?		
Yes	18	9.7
No	167	90.3
Can cats transmit *Toxoplasma* infection?		
Yes	37	20
No	21	11.4
I do not know	127	68.6
Can *Toxoplasma* infection be transmitted by consumption of		
contaminated food or drinks?		
Yes	14	7.6
No	23	12.4
I do not know	148	80
Can *Toxoplasma* infection be transmitted by consumption of		
raw meat?		
Yes	28	15.1
No	10	5.4
I do not know	147	79.5
Can *Toxoplasma* be in meat we eat?		
Yes	25	13.5
No	8	4.3
I do not know	152	82.2
Can *Toxoplasma* be inactivated by freezing meat?		
Yes	11	5.9
No	10	5.4
I do not know	164	88.6
Can *Toxoplasma* infection be transmitted by consumption of		
unboiled or untreated water?		
Yes	18	9.7
No	7	3.8
I do not know	160	86.5
Can *Toxoplasma* infection be transmitted by consumption of		
unwashed fruits or vegetables?		
Yes	21	11.4
No	10	5.4
I do not know	154	83.2
Can *Toxoplasma* infection be transmitted by organ or		
tissue transplantation?		
Yes	9	4.9
No	11	5.9
I do not know	165	89.2
Can *Toxoplasma* infection be transmitted by blood		
transfusion?		
Yes	8	4.3
No	13	7
I do not know	164	88.6
Can *Toxoplasma* be found in cat feces?		
Yes	38	20.5
No	2	1.1
I do not know	145	78.4
Can *Toxoplasma* be found in soil?		
Yes	29	15.7
No	4	2.2
I do not know	152	82.2
Can *Toxoplasma* cause miscarriages?		
Yes	21	11.4
No	2	1.1
I do not know	162	87.6
Can *Toxoplasma* cause disease in fetus?		
Yes	20	10.8
No	3	1.6
I do not know	162	87.6
Have you ever been tested for *Toxoplasma* infection		
during a pregnancy?		
Yes	5	2.7
No	100	54.1
I do not know	65	35.1
Not ever being pregnant	15	8.1
Can *Toxoplasma* cause eye disease?		
Yes	20	10.8
No	6	3.2
I do not know	159	85.9
Do you know how often *Toxoplasma* infection is in the general		
population in Durango City?		
Yes	2	1.1
No	183	98.9
Is there any treatment for toxoplasmosis?		
Yes	7	3.8
No	24	13
I do not know	154	83.2
Do you know someone with toxoplasmosis?		
Yes	2	1.1
No	183	98.9

With respect to practices, studied women had a seniority of 1 to 58 (mean: 17.06 ± 12.77) years, and cooked for 1 to 10 (median: 4) people. Some (4.9%) studied women used to taste raw meat while cooking, and 7.6% used to undercook meat. In addition, 20% of women used to eat raw dried meat, and 13.5% consumed untreated water. Less than 90% of women always washed their hands before cooking, and washed fruits or vegetables. The majority (75.1%) of women never wore gloves when handling raw meat. Whereas, 76.2% of women always washed knife after used for raw meat cutting. About one quarter (27.6%) of women always froze meat. Less than 15% of women allowed animals (cats, dogs, birds) to enter the kitchen. And 16.2% of women cleaned cat feces. Details of the practices found in women are shown in Table 3.

**Table 3:** Practices about toxoplasmosis in the housewives surveyed.

Questions and answers	No.	%
Seniority as a housewife (years)		
Up to 10	76	41.1
11–20	44	23.8
21–30	37	20
31–40	21	11.4
More than 40	7	3.8
For how many people do you cook at home?		
Up to 5	159	85.9
8–10	26	14.1
Do you taste raw meat when cooking?		
Yes	9	4.9
No	176	95.1
How do you cook meat?		
No cook	0	0
Undercook	14	7.6
Well done	171	92.4
How often do you eat raw dried meat?		
Never	148	80
1 to 10 times a year	27	14.6
More than 10 times a year	10	5.4
Do you drink unboiled or untreated water?		
Yes	25	13.5
No	160	86.5
What type of water do you drink at home?		
Unboiled	19	10.3
Bottled	153	82.7
Boiled	9	4.9
Other	4	2.2
How often do you wash your hands before cooking?		
Never	0	0
Sometimes	9	4.9
Almost always	17	9.2
Always	159	85.9
Do you wash fruits before eating?		
Never	0	0
Sometimes	7	3.8
Almost always	14	7.6
Always	164	88.6
Do you wash vegetables before eating?		
Never	2	1.1
Sometimes	3	1.6
Almost always	14	7.6
Always	166	89.7
Do you wear gloves when handling raw meat?		
Never	139	75.1
Sometimes	18	9.7
Almost always	3	1.6
Always	25	13.5
Do you wash a knife used to cut raw meat before use it for another food?		
Never	9	4.9
Sometimes	16	8.6
Almost always	19	10.3
Always	141	76.2
Do you freeze meat?		
Never	59	31.9
Sometimes	46	24.9
Almost always	29	15.7
Always	51	27.6
Do you allow cats to enter to the kitchen?		
Yes	10	5.4
No	41	22.2
No cats at home	134	72.4
Do you allow dogs to enter to the kitchen?		
Yes	26	14.1
No	81	43.8
No dogs at home	78	42.2
Do you have birds in the kitchen?		
Yes	5	2.7
No	180	97.3
Do you clean cat feces?		
Yes	30	16.2
No	155	83.8

The dataset of the study that includes all the data used to obtain the results and conclusions of the study is available in S3 File.

## Discussion

It is important to study housewives regarding knowledge and practices about toxoplasmosis because they prepare meals to their family, and failure in performing hygienic sanitary measures may result in transmission of *T*. *gondii* infection among members of their family. Thus, a housewife is of paramount importance in prevention of infections specially food- and water-borne infection within a family. However, no study regarding knowledge and practices about toxoplasmosis in housewives has been previously reported. Therefore, we determined the knowledge and practices regarding toxoplasmosis in women with housewife occupation in the northern Mexican city of Durango. Results of the present study indicate that studied housewives had a poor knowledge about the parasite, its transmission, the clinical manifestations of the disease, diagnosis, prevention and treatment of infection since less than 10% of surveyed women provided affirmative or correct answers to these items. This finding cannot be compared with others in housewives in other countries because no previous study on knowledge and practices about toxoplasmosis in housewives have been reported. However, a study about knowledge and practices on *Toxoplasma* infection in pregnant women from Malaysia, Philippines, and Thailand reported that only 11% of the studied pregnant women have read, heard, or seen information regarding toxoplasmosis [17]. A very few women knew about the magnitude of *T*. *gondii* infection in the general population in the city. A 6.1% seroprevalence of *T*. *gondii* infection in the general population in Durango City was reported [18]. Only one fifth of the studied women knew about the relation of *T*. *gondii* infection with cats and cat feces. This knowledge should be increased among housewives since 16.2% clean cat feces and therefore, they are at risk for *T*. *gondii* infection and, if they are pregnant, a congenital infection may occur. If the mother is infected for the first-time during pregnancy, she can present a temporary parasitemia that will infect the fetus [19]. Most surveyed women did not have cats at home, but about one fifth of women who had cats at home allowed them to enter the kitchen. Cooking is one of the routine activities of housewives, but a lack of knowledge about the preventive measures against infection regarding hygiene of food and water, degree of meat cooking, and inactivation of the parasite may lead to wrong practices that may favor transmission of infection among family members. In fact, only 7.6% of women knew about the risk of infection with *T*. *gondii* by consumption of contaminated food or water. About one of ten women did not always washed fruits or vegetables or wash hands before cooking. Results reflect poor hygiene practices among a considerable number of studied housewives. Thus, education of housewives to improve their hygiene practices when preparing food and avoid *T*. *gondii* infection is needed. Seropositivity to *T*. *gondii* has been associated with consumption of unwashed raw fruits [20], and unwashed raw vegetables [21] in Durango City. An important number of women ate raw dried meat (20%), tasted raw meat when cooking (4.9%), and undercooked meat (7.6%). An association between *T*. *gondii* seropositivity and consumption of dried meat was reported in the USA [22]. In addition, consumption of raw or undercooked meat was associated with *T*. *gondii* seropositivity in Durango City [20, 23]. A minority of women wore gloves when handling raw meat and this might increase the risk for *T*. *gondii* infection specially if hands are not washed after this handling. A considerable number (23.8%) of women did not always washed the knife after used for raw meat cutting. This practice might represent a risk for contamination of food. Freezing raw meat can kill *T*. *gondii* [24]. However, only about one quarter of the surveyed women always froze meat. Consumption of untreated water is a risk for *T*. *gondii* infection. In a study in Mennonites in rural Durango, Mexico, consumption of untreated water was associated with *T*. *gondii* seropositivity [25]. In the present study, the finding of consumption of untreated water in housewives is of concern and suggests that this practice might also occur in other members of their family.

We have been studying the knowledge and practices about toxoplasmosis in populations in Durango, Mexico. In a first study, we examined physicians attending pregnant women and found an incomplete knowledge about diagnosis and treatment of toxoplasmosis [26]. In a second study, we studied clinical laboratory professionals and found also an incomplete knowledge of *T*. *gondii* infection and toxoplasmosis and a limited practice of laboratory tests among the professionals surveyed [27]. Results of the present study add information of the knowledge and practices about toxoplasmosis in populations in Mexico. Results of these studies reflect a suboptimal knowledge about toxoplasmosis in the studied populations, and education about this topic to improve the health of the population is needed.

The present survey used a questionnaire that can be modified for studying the knowledge and practices about toxoplasmosis in housewives in other countries including for instance, items about sociodemographic data, treatment, and prevalence.

## Conclusions

This is the first study regarding knowledge and practices about toxoplasmosis in housewives. Poor knowledge regarding *T*. *gondii* infection, toxoplasmosis, and practices to avoid infection among the housewives studied was found. High risk practices for infection were identified as consumption of dried raw meat, undercooked meat, and untreated water. Strategies to improve toxoplasmosis-related knowledge and practices to avoid *T*. *gondii* infection and its sequelae in housewives are highly needed.

## Supporting information

S1 File Questionnaire in Spanish. (PDF)

S2 File Questionnaire in English. (PDF)

S3 File Date set of the study. (XLSX)
