# Supplementary material for: Knowledge and practices regarding toxoplasmosis in housewives: A cross sectional study in a northern Mexican city
Source: PLoS One. 2019 Sep 9;14(9):e0222094. doi: 10.1371/journal.pone.0222094 (PMC6733448; doi:10.1371/journal.pone.0222094)
Supplement: S1 File — (PDF) [file pone.0222094.s001.pdf]

Cuestionario – *Tg* - Amas de casa

Folio:

Edad:

Sección I

1) Lugar de nacimiento:
   (1) Durango, México
   (2) Otro Estado de México. ¿Cuál?
   (3) Otro país. ¿Cuál?

2) Lugar de residencia:
   (1) Durango, México
   (2) Otro Estado de México. ¿Dónde?
   (3) En el extranjero. ¿Dónde?

3) Área de residencia:
   (1) Urbana (2) Suburbana (3) Rural

4) Escolaridad:
   (1) No estudios (0 años)
   (2) Primaria (1-6 años)
   (3) Secundaria o preparatoria (7-12 años)
   (4) Profesional o posgrado (13 o más años)

5) Nivel socioeconómico: (1) Bajo (2) Medio (3) Alto

Sección II.

6) ¿Cuántos años lleva como ama de casa?

7) ¿Para cuantas personas cocina en su casa?

8) ¿Prueba carne cruda cuando cocina?
   (1) Sí (2) No

9) ¿A qué punto coce la carne?
   (1) No la coce (Cruda) (2) Poco cocida (3) Bien cocida.

10) ¿Qué tan frecuentemente comen carne seca cruda?
   (1) Nunca
   (2) Pocas veces (de 1 a 10 veces por año)
   (3) Frecuentemente (más de 10 veces al año)

11) ¿Consumen agua no tratada (no hervida o no purificada)?
   (1) Sí (2) No

12) ¿Qué tipo(s) de agua usan para tomar?

(1) De la llave sin hervir
(2) De garrafón
(3) Hervida
(4) De otra, ¿cuál?

13) ¿Qué tan frecuentemente se lava las manos antes de preparar los alimentos?

(1) Nunca
(2) A veces
(3) Casi siempre
(4) Siempre

14) ¿Lava la fruta antes de comerla?

(1) Nunca
(2) A veces
(3) Casi siempre
(4) Siempre

15) ¿Lava la verdura antes de comerla?

(1) Nunca
(2) A veces
(3) Casi siempre
(4) Siempre

16) ¿Usa guantes cuando manipula la carne cruda?

(1) Nunca
(2) A veces
(3) Casi siempre
(4) Siempre

17) ¿Lava el cuchillo con el que cortó carne cruda antes de cortar otros alimentos?

(1) Nunca
(2) A veces
(3) Casi siempre
(4) Siempre

18) ¿Congela la carne?

(1) Nunca
(2) A veces
(3) Casi siempre
(4) Siempre

19) En caso de tener gatos, ¿permite que los gatos entren a la cocina?

(1) Sí (2) No (3) No tiene gatos

20) En caso de tener perros, ¿permite que los perros entren a la cocina?
(1) Sí (2) No (3) No tiene perros

21) ¿Tiene pajaritos en la cocina?
(1) Sí (2) No

22) ¿Limpia usted los excrementos de gatos?
(1) Sí (2) No

Sección III.
23) ¿Sabe que es *Toxoplasma gondii*?
(1) Sí ¿Qué es? (2) No

24) ¿Sabe que es la toxoplasmosis?
(1) Sí ¿Qué es? (2) No

25) ¿Sabe cómo se puede transmitir la infección por *Toxoplasma gondii*?
(1) Sí ¿Cómo? (2) No

26) ¿Sabe que manifestaciones clínicas se presentan en la toxoplasmosis?
(1) Sí ¿Cuáles son? (2) No

27) ¿Sabe cómo se diagnostica la infección por *Toxoplasma gondii*?
(1) Sí ¿Cómo? (2) No

28) ¿Sabe cómo evitar la toxoplasmosis?
(1) Sí ¿Cómo? (2) No

29) ¿Los gatos pueden transmitir la infección por *Toxoplasma gondii*?
(1) Sí (2) No (3) No sé

30) ¿La infección por *Toxoplasma gondii* se puede transmitir por consumir alimentos o bebidas contaminadas?
(1) Sí ¿Cuáles? (2) No (3) No sé

31) ¿La infección por *Toxoplasma gondii* se puede transmitir por comer carne cruda?
(1) Sí (2) No (3) No sé

32) ¿Puede encontrarse *Toxoplasma gondii* en la carne que consumimos?
(1) Sí (2) No (3) No sé

33) ¿Puede inactivarse *Toxoplasma gondii* congelando la carne?
(1) Sí (2) No (3) No sé

34) ¿La infección por *Toxoplasma gondii* se puede transmitir por tomar agua no hervida o no tratada?

(1) Sí (2) No (3) No sé

35) ¿La infección por *Toxoplasma gondii* se puede transmitir por comer fruta o verdura cruda no lavada?

(1) Sí (2) No (3) No sé

36) ¿La infección por *Toxoplasma gondii* se puede transmitir por los trasplantes de órganos o tejidos?

(1) Sí (2) No (3) No sé

37) ¿La infección por *Toxoplasma gondii* se puede transmitir por trasfusiones de sangre?

(1) Sí (2) No (3) No sé

38) ¿*Toxoplasma gondii* se puede encontrar en el excremento de los gatos?

(1) Sí (2) No (3) No sé

39) ¿*Toxoplasma gondii* puede estar en el suelo?

(1) Sí (2) No (3) No sé

40) ¿*Toxoplasma gondii* puede causar abortos?

(1) Sí (2) No (3) No sé

41) ¿*Toxoplasma gondii* puede causar enfermedad en el feto?

(1) Sí (2) No (3) No sé

42) ¿Le han hecho la prueba para detectar la infección por *Toxoplasma gondii* en algún embarazo?

(1) Sí (2) No (3) No sé (4) No se ha embarazado

43) ¿*Toxoplasma gondii* puede causar enfermedad de la vista?

(1) Sí (2) No (3) No sé

44) ¿Sabe que tan frecuentemente ocurre la infección por *Toxoplasma gondii* en la población general de la ciudad de Durango?

(1) Sí ¿Qué porcentaje? (2) No

45) ¿Hay tratamiento para la toxoplasmosis?

(1) Sí ¿Cuál? (2) No (3) No sé

46) ¿Ha conocido a alguien con toxoplasmosis?

(1) Sí ¿Quién? (2) No
