# Supplementary material for: Knowledge and practices regarding toxoplasmosis in housewives: A cross sectional study in a northern Mexican city
Source: PLoS One. 2019 Sep 9;14(9):e0222094. doi: 10.1371/journal.pone.0222094 (PMC6733448; doi:10.1371/journal.pone.0222094)
Supplement: S2 File — (PDF) [file pone.0222094.s002.pdf]

Questionnaire – *Tg* - Housewives

Number:

Age:

Section I

1) Birthplace:
   (1) Durango, Mexico
   (2) Another Mexican state. Which?
   (3) Other country. Which?

2) Residence place:
   (1) Durango, Mexico
   (2) Another Mexican state. Where?
   (3) Abroad. Where?

3) Residence area:
   (1) Urban (2) Suburban (3) Rural

4) Education:
   (1) No education (0 years)
   (2) Primary school (1-6 years)
   (3) High school (7-12 years)
   (4) Graduate or postgraduate (13 or more years)

5) Socioeconomic status: (1) Low (2) Medium (3) High

Section II.

6) Seniority as a housewife (years):

7) For how many people do you cook at home?

8) Do you taste raw meat when cooking?
   (1) Yes (2) No

9) How do you cook meat?
   (1) No cook (raw) (2) Undercooked (3) Well done

10) How often do you eat raw dried meat?
   (1) Never
   (2) One to 10 times a year
   (3) More than 10 times a year

11) Do you drink unboiled or untreated water?
   (1) Yes (2) No

12) What type of water do you drink at home?
(1) Unboiled
(2) Bottled
(3) Boiled
(4) Another. Which?

13) How often do you wash your hands before cooking?
(1) Never
(2) Sometimes
(3) Almost always
(4) Always

14) Do you wash fruits before eating?
(1) Never
(2) Sometimes
(3) Almost always
(4) Always

15) Do you wash vegetables before eating?
(1) Never
(2) Sometimes
(3) Almost always
(4) Always

16) Do you wear gloves when handling raw meat?
(1) Never
(2) Sometimes
(3) Almost always
(4) Always

17) Do you wash a knife used to cut raw meat before use it for another food?
(1) Never
(2) Sometimes
(3) Almost always
(4) Always

18) Do you freeze meat?
(1) Never
(2) Sometimes
(3) Almost always
(4) Always

19) If you have cats, do you allow cats to enter to the kitchen?
(1) Yes (2) No (3) No cats at home

20) If you have dogs, do you allow dogs to enter to the kitchen?

(1) Yes (2) No (3) No dogs at home

21) Do you have birds in the kitchen?
(1) Yes (2) No

22) Do you clean cat feces?
(1) Yes (2) No

Section III.
23) Do you know what is *Toxoplasma gondii*?
(1) Yes. What is it? (2) No

24) Do you know what toxoplasmosis is?
(1) Yes. What is it? (2) No

25) Do you know how *Toxoplasma gondii* is transmitted?
(1) Yes. How? (2) No

26) Do you know the clinical manifestations of toxoplasmosis?
(1) Yes. What are they? (2) No

27) Do you know how *Toxoplasma infection* is diagnosed?
(1) Yes. How? (2) No

28) Do you know how to avoid toxoplasmosis?
(1) Yes. How? (2) No

29) Can cats transmit *Toxoplasma* infection?
(1) Yes (2) No (3) I do not know

30) Can *Toxoplasma* infection be transmitted by consumption of contaminated food or drinks?
(1) Yes. Which? (2) No (3) I do not know

31) Can *Toxoplasma* infection be transmitted by consumption of raw meat?
(1) Yes (2) No (3) I do not know

32) Can *Toxoplasma* be in meat we eat?
(1) Yes (2) No (3) I do not know

33) Can *Toxoplasma* be inactivated by freezing meat?
(1) Yes (2) No (3) I do not know

34) Can *Toxoplasma* infection be transmitted by consumption of unboiled or untreated water?
(1) Yes (2) No (3) I do not know

35) Can *Toxoplasma* infection be transmitted by consumption of unwashed fruits or vegetables?
(1) Yes (2) No (3) I do not know

36) Can *Toxoplasma* infection be transmitted by organ or tissue transplantation?
(1) Yes (2) No (3) I do not know

37) Can *Toxoplasma* infection be transmitted by blood transfusion?
(1) Yes (2) No (3) I do not know

38) Can *Toxoplasma* be found in cat feces?
(1) Yes (2) No (3) I do not know

39) Can *Toxoplasma* be found in soil?
(1) Yes (2) No (3) I do not know

40) Can *Toxoplasma* cause miscarriages?
(1) Yes (2) No (3) I do not know

41) Can *Toxoplasma* cause disease in fetus?
(1) Yes (2) No (3) I do not know

42) Have you ever been tested for *Toxoplasma* infection during a pregnancy?
(1) Yes (2) No (3) I do not know (4) Not ever been pregnant

43) Can *Toxoplasma* cause eye disease?
(1) Yes (2) No (3) I do not know

44) Do you know how often *Toxoplasma* infection is in the general population in Durango City?
(1) Yes. What percentage? (2) No

45) Is there any treatment for toxoplasmosis?
(1) Yes. Which? (2) No (3) I do not know

46) Do you know someone with toxoplasmosis?
(1) Yes. Who? (2) No
